# First two mitochondrial genomes for the order *Filobasidiales* reveal novel gene rearrangements and intron dynamics of *Tremellomycetes*

**DOI:** 10.1186/s43008-022-00094-2

**Published:** 2022-05-02

**Authors:** Qiang Li, Zhijie Bao, Ke Tang, Huiyu Feng, Wenying Tu, Lijiao Li, Yunlei Han, Mei Cao, Changsong Zhao

**Affiliations:** 1 Key Laboratory of Coarse Cereal Processing, Ministry of Agriculture and Rural Affairs, School of Food and Biological Engineering, Chengdu University, Chengdu, Sichuan China; 2 Present Address: School of Public Health, Chengdu Medical College, Chengdu, Sichuan China; 3 Core Laboratory, Sichuan Provincial People’s Hospital, University of Electronic Science and Technology of China, Chengdu, Sichuan China; 4 Chinese Academy of Sciences Sichuan Translational Medicine Research Hospital, Chengdu, Sichuan China; 5 Department of Pathogenic Biology, Chengdu Medical College, Chengdu, Sichuan China

**Keywords:** *Tremellomycetes*, Mitochondrial genome, Intron, Gene rearrangement, Evolution, Phylogenetic analysis

## Abstract

In the present study, two mitogenomes from the *Filobasidium* genus were assembled and compared with other *Tremellomycetes* mitogenomes. The mitogenomes of *F. wieringae* and *F. globisporum* both comprised circular DNA molecules, with sizes of 27,861 bp and 71,783 bp, respectively. Comparative mitogenomic analysis revealed that the genetic contents, tRNAs, and codon usages of the two *Filobasidium* species differed greatly. The sizes of the two *Filobasidium* mitogenomes varied greatly with the introns being the main factor contributing to mitogenome expansion in *F. globisporum*. Positive selection was observed in several protein-coding genes (PCGs) in the *Agaricomycotina, Pucciniomycotina*, and *Ustilaginomycotina* species, including *cob*, *cox2*, *nad2*, and *rps3* genes. Frequent intron loss/gain events were detected to have occurred during the evolution of the *Tremellomycetes* mitogenomes, and the mitogenomes of 17 species from *Agaricomycotina*, *Pucciniomycotina*, and *Ustilaginomycotina* have undergone large-scale gene rearrangements. Phylogenetic analyses based on Bayesian inference and the maximum likelihood methods using a combined mitochondrial gene set generated identical and well-supported phylogenetic trees, wherein *Filobasidium* species had close relationships with *Trichosporonales* species. This study, which is the first report on mitogenomes from the order *Filobasidiales*, provides a basis for understanding the genomics, evolution, and taxonomy of this important fungal group.

**Supplementary Information:**

The online version contains supplementary material available at 10.1186/s43008-022-00094-2.

## INTRODUCTION

Mitochondria are important organelles of fungi that play important roles in fungal growth and environmental adaptation (Ernster and Schatz 1981; McBride et al. 2006; Murphy 2009). Fungal mitochondria contain their own genomes, which are considered the “second genomes” of fungi (mitogenome). Fungal mitogenomes have several characteristics that differ from nuclear genomes, including a high copy number in one fungal cell, a smaller size relative to nuclear genomes, and uniparental inheritance in most species (Basse 2010). Most fungi contain a set of core protein coding genes, including *atp6*, *atp8*, *atp9*, *cob*, *cox1*, *cox2*, *cox3*, *nad1*, *nad2*, *nad3*, *nad4*, *nad4L*, *nad5*, *nad6*, and *rps3* (Li et al. 2021a). Mitochondrial genes play important roles in maintenance of cell homeostasis and cell energy supplies as well as regulation of fungal growth (Chatre and Ricchetti 2014; Osiewacz 2002). In addition, variations and evolution of the mitogenome have become effective molecular markers reflecting the phylogenetic status and relationships among fungal species (Cheng et al. 2021; Li et al. 2018b, b). However, the number of basidiomycetes mitogenomes available in the National Center for Biotechnology Information (NCBI) database is currently far lower than that of animals (https://www.ncbi.nlm.nih.gov/genome/browse#!/organelles/). Indeed, although there are about 1 million species of basidiomycetes, there are less than 120 basidiomycetes mitogenomes available in the database. Basidiomycetes are widely distributed worldwide and play important roles in industry, medicine, agriculture, food supply, and maintenance of forest ecosystems (Alves et al. 2013; Elisashvili 2012; Voriskova and Baldrian 2013). Accordingly, analysis of basidiomycete mitogenomes will help us understand their origin and evolution and lay a foundation for better utilization of basidiomycetes.

*Tremellomycetes*, belonging to *Agaricomycotina*, is a class of fungi with multiple morphological characteristics (Hibbett 2006; Liu et al. 2015a). To date, hundreds of *Tremellomycetes* species have been described, including yeasts, dimorphic taxa, and species that form hyphae and/or complex basidiocarps (Millanes et al. 2011; Yurkov and Kurtzman 2019). Classification of *Tremellomycetes* based on phenotype usually leads to confusion or inaccuracy; however, the introduction of molecular markers has improved our understanding of the phylogenetic relationships among *Tremellomycetes* (Liu et al. 2015b; Wang and Wang 2015). The genus *Filobasidium* is an important group of *Tremellomycetes* found in plant leaves, soil and water (Li et al. 2020a; Nemcova et al. 2015). Some species of *Filobasidium* have been found to be opportunistic pathogens, some have excellent environmental tolerance, and some are antimicrobial (Luo et al. 2019; Pan et al. 2012; Singh et al. 2013). Because of their diverse physiological characteristics and lifestyles, members of the genus *Filobasidium* comprise one of the most important groups for analysis of the evolution of *Tremellomycetes* species. Nevertheless, no mitogenomes from the genus *Filobasidium* or even the order *Filobasidiales* have been published to date.

In the present study, the mitogenomes of two *Filobasidium* species, *Filobasidium wieringae*, and *Filobasidium globisporum*, were assembled and compared. The aim of this study was to (1) reveal the genetic characteristics of *Filobasidium* mitogenomes; (2) characterize the evolutionary dynamics of introns in *Tremellomycetes* mitogenomes; and (3) determine the phylogenetic placement of *Filobasidium* in the phylum Basidiomycota based on the combined mitochondrial gene dataset (15 core PCGs). This is the first report of *Filobasidium* mitogenomes*.* The availability of these mitogenomes will improve our understanding of the genetics, evolution, and taxonomy of *Filobasidium* species and other closely related fungal species.

## MATERIALS AND METHODS

### Mitogenome assembly and annotation

The raw sequencing data used for *F. wieringae* and *F. globisporum* mitogenomes assembly were downloaded from the Sequence Read Archive database (accession numbers SRR4171232 and DRR032577, respectively). Quality control steps conducted to remove unqualified sequences from the raw sequencing data consisted of filtering low-quality sequences using ngsShoRT 2.2 (Chen et al. 2014) and removing adapter reads using AdapterRemoval v2 (Schubert et al. 2016). The clean reads were then applied to assemble the mitogenomes of *Filobasidium* species using NOVOPlasty v4.2.1 with a K-mer size of 29 (Dierckxsens et al. 2017). The circularized assembled mitogenomes of the two *Filobasidium* species were annotated as previously described (Li et al. 2018a). Briefly, the open reading frames (ORFs), protein-coding genes (PCGs), rRNAs, tRNAs, and introns in the two *Filobasidium* mitogenomes were initially annotated using M Fannot (Valach et al. 2014) and MITOS (Bernt et al. 2013), which are both based on the mitochondrial genetic code 4. ORFs (> 100 aa) were further modified based on the NCBI Open Reading Frame Finder (Coordinators 2017) and annotated by BLASTP searches against the NCBI non-redundant protein sequence database (Bleasby and Wootton 1990). We determined the intron and exon boundaries in the ORFs using exonerate v2.2 (Slater and Birney 2005). tRNA genes in the two mitogenomes were predicted using the tRNA scan-SE v1.3.1 software (Lowe and Chan 2016). Physical maps of the two *Filobasidium* mitogenomes were generated using OGDraw v1.2 (Lohse et al. 2013). In the maps, the inner grayscale bar graph shows the GC content of the mitochondrial sequences and the circles inside the GC content graph mark the 50% threshold.

### Sequence analysis

Base compositions of the two *Filobasidium* mitogenomes and other mitogenomes were analyzed using DNASTAR Lasergene v7.1 (http://www.dnastar.com/). Strand asymmetries of *Tremellomycetes* mitogenomes were calculated based on the following formulas: AT skew = [A − T]/[A + T], and GC skew = [G − C]/[G + C]. Codon usages of the two *Filobasidium* mitogenomes were analyzed using Sequence Manipulation Suite (Stothard 2000). The nonsynonymous (*Ka*) and synonymous (*Ks*) substitution rates for core PCGs in the 17 mitogenomes from *Agaricomycotina*, *Pucciniomycotina*, and *Ustilaginomycotina* were calculated using DnaSP v6.10.01 (Rozas et al. 2017). The genetic distances between each pair of the 15 core PCGs (*atp6, atp8, atp9, cob*, *cox1, cox2, cox3, nad1, nad2, nad3, nad4, nad4L, nad5, nad6,* and *rps3*) in the 17 mitogenomes were analyzed using MEGA v6.06 based on the Kimura-2-parameter (K2P) substitution model (Caspermeyer 2016). To determine if there were intra-genomic duplications of large fragments or interspersed repeats in the two *Filobasidium* mitogenomes, we conducted BlastN searches (e-value < 10^−10^) of the two *Filobasidium* mitogenomes against themselves (Chen et al. 2015). Tandem repeats (> 10 bp in length) in the two *Filobasidium* mitogenomes were detected using the Tandem Repeats Finder (Benson 1999) with the default parameters.

### Comparative mitogenomic analysis and intron analysis

Genome sizes, GC contents, base compositions, and gene and intron numbers of the 17 mitogenomes from *Agaricomycotina*, *Pucciniomycotina*, and *Ustilaginomycotina* were compared to assess variations and conservations among different mitogenomes. We further calculated the contribution rate of different regions to the size variations of the two *Filobasidium* mitogenomes using the following formula: size difference of region/size difference of the entire mitogenome *100%. We classified introns of *cox1* genes in the 17 mitogenomes into different position classes (Pcls) according to the method described by Férandon et al. (Ferandon et al. 2010). We first aligned the *cox1* genes of the 17 mitogenomes with the *cox1* gene of *Ganoderma calidophilum* (Li et al. 2019d), which was used as a reference in previous studies (Ye et al. 2020) using Clustal W (Thompson et al. 1994). Introns inserted at the same position of the *cox1* reference gene belonging to the same Pcl, which were named according to the insert sites (nt) in the corresponding reference gene. When the same Pcls are present, they are considered orthologous introns and usually have high sequence similarity (Ferandon et al. 2010).

### Phylogenetic analysis

A phylogenetic tree of 79 Basidiomycota species was constructed using the combined mitochondrial gene set (15 core PCGs) to investigate the phylogenetic status of *Filobasidium* species in the phylum Basidiomycota. The phylogenetic tree was constructed as previously described (Cheng et al. 2021; Li et al. 2020b). *Annulohypoxylon stygium* from the phylum *Ascomycota* was used as the outgroup (Deng et al. 2018). We first aligned individual mitochondrial genes using MAFFT v7.037 (Katoh et al. 2019), after which we concatenated these aligned mitochondrial sequences into a combined mitochondrial gene set using the Sequence Matrix v1.7.8 (Vaidya et al. 2011). A preliminary partition homogeneity test was conducted to detect potential phylogenetic conflicts between different mitochondrial genes. Partition Finder 2.1.1 (Lanfear et al. 2017) was used to detect best-fit models of partitioning schemes and evolution for the combined mitochondrial gene set. Phylogenetic trees were constructed using both the Bayesian inference (BI) and maximum likelihood (ML) methods. We conducted BI analysis using MrBayes v3.2.6 (Ronquist et al. 2012). Two independent runs with four chains (three heated and one cold) each were conducted simultaneously for 2 × 10^6^ generations. Each run was sampled every 100 generations. We assumed that stationarity had been reached when the estimated sample size was greater than 100 and the potential scale reduction factor approached 1.0. The first 25% of samples were discarded as burn-in, and the remaining trees were used to calculate Bayesian posterior probabilities (BPP) in a 50% majority-rule consensus tree (Wu et al. 2021). RAxML v 8.0.0 (Stamatakis 2014) was used to conduct the ML analysis. Bootstrap values (BS) were assessed through an ultrafast bootstrap approach with 1000 replicates.

### Data availability

The complete mitogenomes of *F. wieringae* and *F. globisporum* were deposited in the GenBank database (Benson et al. 2018) under accession numbers MW039344 and MW039345, respectively.

## RESULTS

### Characterization and PCGs of* Filobasidium mitogenomes*

**Fig. 1:**
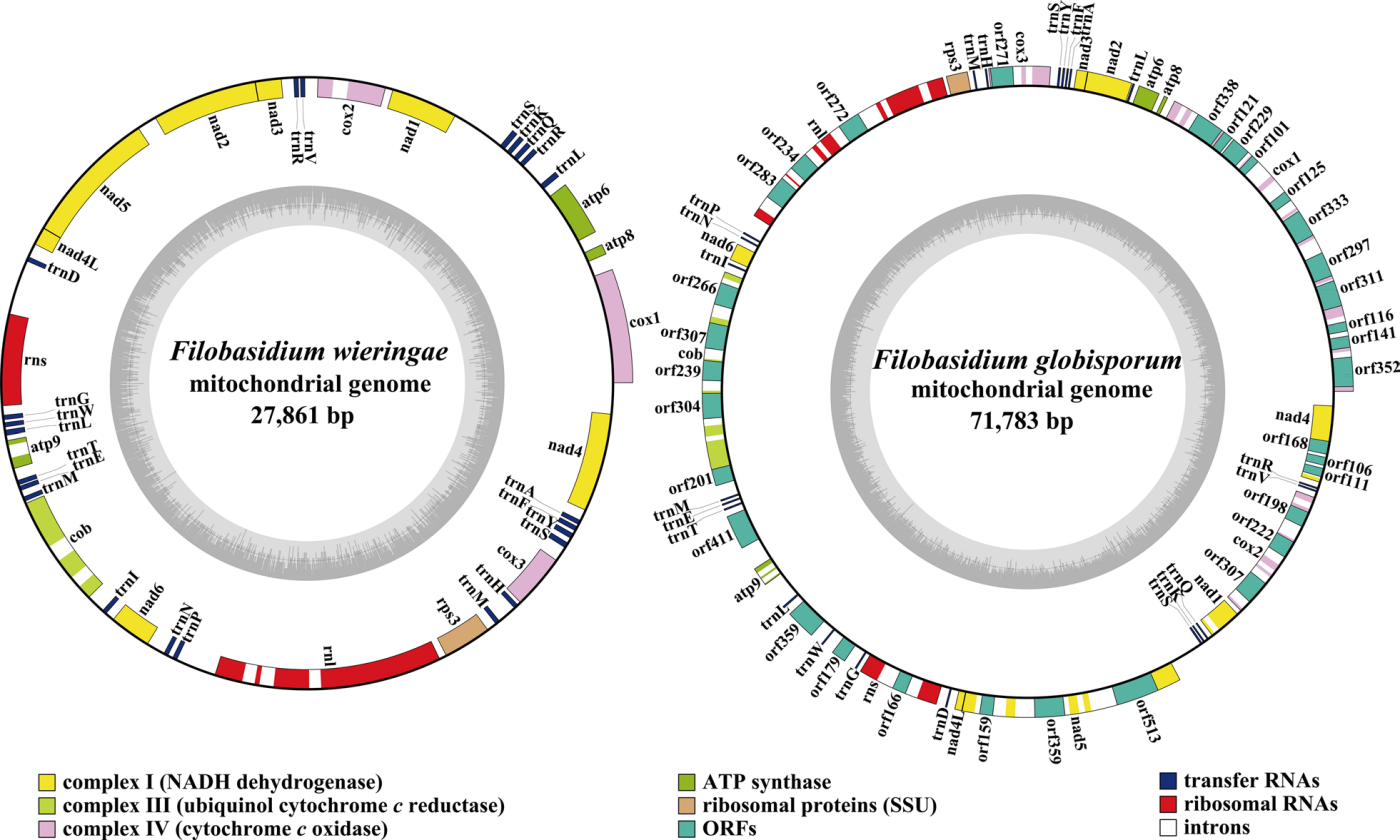
Circular maps of the mitochondrial genomes of two *Filobasidium* species. Genes are represented by different colored blocks. Colored blocks outside each ring indicate that the genes are on the direct strand, while colored blocks within the ring indicates that the genes are located on the reverse strand. The inner grayscale bar graph shows the GC content of the mitochondrial sequences. The circle inside the GC content graph marks the 50% threshold

The structures of the two *Filobasidium* mitogenomes were circular, with total lengths of 27,861 bp and 71,783 bp for *F. wieringae* and *F. globisporum*, respectively (Fig. 1). The complete mitogenomes of *F. wieringae* and *F. globisporum* had GC contents of 38.51% and 40.32%, respectively (Additional file 1: Table S1). The mitogenome of *F. wieringae* had a negative AT skew and positive GC skew while the mitogenome of *F. globisporum* contained positive AT and negative GC skews. A total of 15 and 48 PCGs were detected in the mitogenomes of *F. wieringae* and *F. globisporum*, respectively. Both the mitogenomes contained a set of core PCGs shared by basidiomycete mitogenomes, including *atp6*, *atp8*, *atp9*, *cob*, *cox1*, *cox2*, *cox3*, *nad1*, *nad2*, *nad3*, *nad4*, *nad4L*, *nad5*, *nad6*, and *rps3* (Tables S2 and S3). In addition, the mitogenome of *F. globisporum* contained four non-conserved PCGs encoding proteins with unknown functions and three non-conserved PCGs encoding GIY endonucleases. The *F. globisporum* mitogenome contained 37 introns distributed in the *atp9*, *cob*, *cox1*, *cox2*, *cox3*, *nad1*, *nad4*, *nad5*, *rns*, and *rnl* genes, of which 27 belonged to group I, two belonged to group II, and eight were of unknown types. A total of 26 intronic ORFs were detected in these introns, encoding LAGLIDADG homing endonucleases, GIY-YIG homing endonucleases, and proteins with unknown functions. Seven introns were detected in the mitogenome of *F. wieringae*, six of which belonged to group I. No intronic ORFs were detected in introns of *F. wieringae*.

### rRNA and tRNA genes in the *Filobasidium mitogenomes*

Both *Filobasidium* mitogenomes contained two rRNA genes, namely the small subunit ribosomal RNA (*rns*) and the large subunit ribosomal RNA (*rnl*) (Additional file 1: Table S2). The *rnl* gene of *F. globisporum* was 36 bp longer than that of *F. wieringae*. The two *Filobasidium* mitogenomes contained identical length *rns* genes. The mitogenomes of *F. wieringae* and *F. globisporum* contained 23 and 22 tRNA genes, respectively, which were all folded into classical cloverleaf structures (Additional file 2: Fig. S1). The two mitogenomes contained two tRNAs with different anticodons coding for serine and leucine. The mitogenome of *F. wieringae* also contained 2 tRNAs with different anticodons encoding Arginine. The size of individual tRNAs ranged from 71 to 86 bp, mainly due to size variations of the extra arms. Of the 22 tRNA genes shared by the two *Filobasidium* mitogenomes, 17 contained sites that varied between the two mitogenomes. A total of 159 variable sites were detected in the 22 tRNA genes between the two *Filobasidium* mitogenomes. The most common variable site was located on the extra arm (36 sites varied between the two mitogenomes), followed by the D arm.

### Overlapping nucleotides and composition of mitogenomes

Two overlapping nucleotides were detected in the mitogenome of *F. wieringae* across the neighboring genes *nad2* and *nad3* (− 1 bp), as well as between *nad4L* and *nad5* (− 1 bp) (Additional file 1: Table S2). We detected three sets of overlapping nucleotides in the mitogenome of *F. globisporum*, with the largest set located between *cox3* and orf201 (− 19 bp). A total of 6870 bp and 10,849 bp of intergenic sequences were detected in the mitogenomes of *F. wieringae* and *F. globisporum*, respectively. The length of these intergenic sequences ranged from 16 to 1291 bp. The longest intergenic sequence was located between *nad5* and *trns* in the *F. globisporum* mitogenome.

**Fig. 2:**
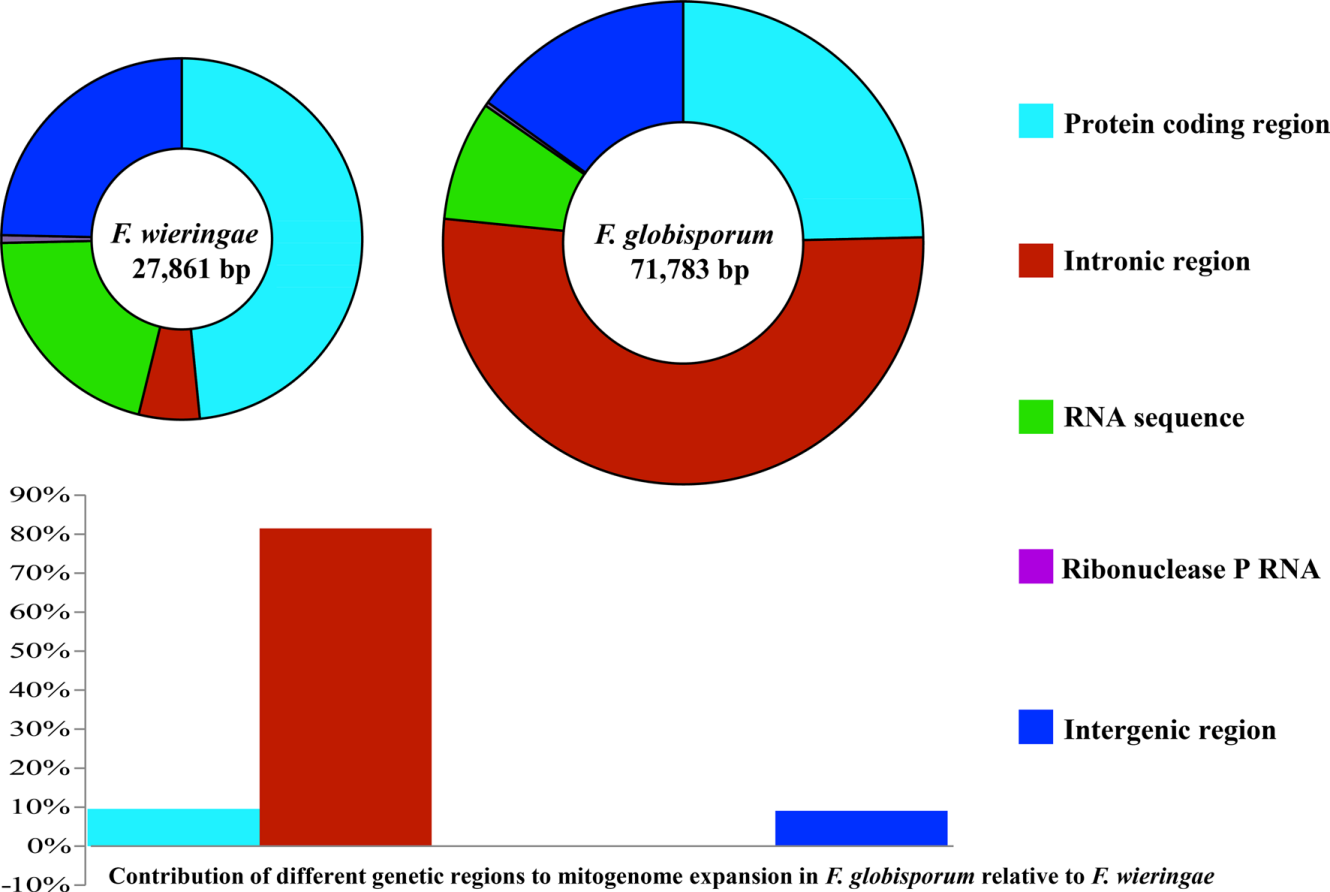
Mitogenome composition of the entire mitochondrial genomes of the two *Filobasidium* species. The bottom panel shows the contribution of different gene regions to the expansion of the *F. globisporum* mitogenome. The y-axis represents the contribution rate of different regions to the size variation of the whole mitogenome, which is calculated by the following formula: size difference of region / size different of the entire mitogenome *100%

The protein coding regions accounted for the largest proportion of the *F. wieringae* mitogenome (48.40%) (Fig. 2), while the intronic regions accounted for the largest proportion of the *F. globisporum* mitogenome, occupying 51.96%. Intergenic regions occupied 15.11–24.66% of the two mitogenomes, while ribonuclease P RNA coding regions accounted for the smallest proportion of the two mitogenomes (0.26%–0.69%). The *F. wieringae* mitogenome was 43,922 bp smaller than that of *F. globisporum*. Intronic regions made the greatest contribution to the size expansion of the *F. globisporum* mitogenome, with a contributing rate of 81.48%. Protein coding regions accounted for 9.56% of the size variation, while intergenic regions contributed 9.06% of the *F. globisporum* mitogenome expansion.

### Codon usage analysis

Most of the core PCGs in *Agaricomycotina, Pucciniomycotina*, and *Ustilaginomycotina* mitogenomes used ATG as the start codon, except for the *cox1* gene of *Ustilago bromivora* and the *cox1* and *nad2* genes of *Ustilago maydis*, which used GTG, and the *cox2* and *rps3* genes of *Jaminaea angkorensis*, which used TTG (Additional file 1: Table S4). TAA was the most widely used stop codon in the core PCGs of the 17 mitogenomes tested, followed by TAG. We found that the start and stop codons varied greatly between *Tremellomycetes* species, even those that were closely related. The *atp9* gene of *F. wieringae* used TAG as the stop codon, while that of *F. globisporum* used TAA as the stop codon. The *cob*, *cox2*, *nad4*, and *nad5* genes of *F. globisporum* used TAG as stop codons, while those of *F. wieringae* used TAA as stop codons. Within *Ustilaginomycotina*, the *nad2* gene of *Ustilago maydis* used GTG as the start codon, while *Ustilago bromivora* used ATG as the start codon.

**Fig. 3:**
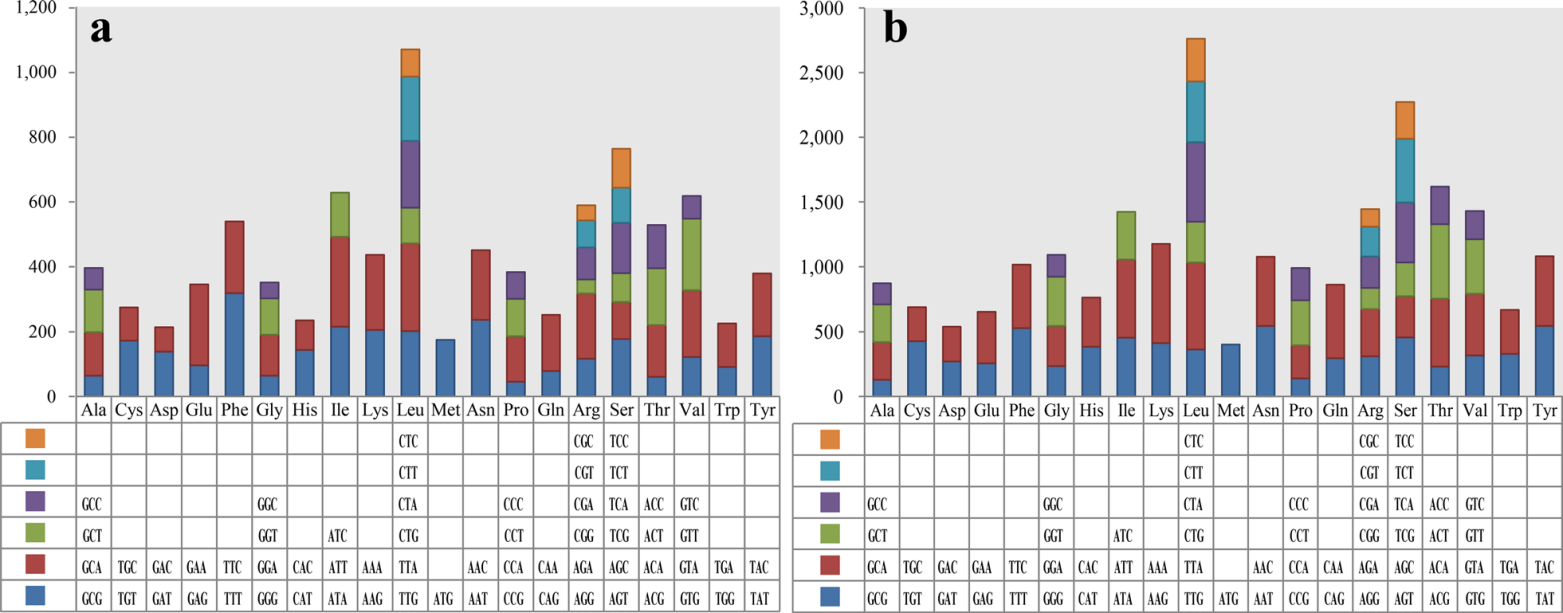
Codon usage in the mitochondrial genomes of two *Filobasidium* species. Frequency of codon usage is plotted on the y-axis. **a**, *F. wieringae*; **b***, F. globisporum*

Codon usage analysis indicated that the most frequently used codons in the two *Filobasidium* mitogenomes were CAA (for glutamine; Gln), GAA (for glutamic acid; Glu), TGT (for cysteine; Cys), AAA (for lysine; Lys), GAT (for aspartic acid; Asp), and CAT (for histidine; His) (Fig. 3). The frequent use of A and T in codons contributed to a relatively high AT content in the two *Filobasidium* mitogenomes (average: 60.59%).

### Repetitive sequences analysis

We conducted BLASTn searches of the two *Filobasidium* mitogenomes against themselves and identified 0 and 16 repeat sequences in the mitogenomes of *F. wieringae* and *F. globisporum*, respectively (Additional file 1: Table S5). The length of repeat sequences in the *F. globisporum* mitogenome ranged from 44 to 695 bp, with pair-wise nucleotide similarities ranging from 71.66 to 95.46%. The largest repeats were located in the fourth intron and fifth exons of the *nad5* gene in the *F. globisporum* mitogenome. The second largest repeats were located in the intergenic region between *trnR* and *orf111*, as well as in the exon and intron regions of the *nad4* gene in the *F. globisporum* mitogenome, with a repeating sequence 165 bp long. Repeat sequences accounted for 3.74% of the *F. globisporum* mitogenome. Both *Filobasidium* mitogenomes contained two tandem repeats (Additional file 1: Table S6). The longest tandem repeat sequence (66 bp) was detected in the intergenic region between *rnl* and *trnP* in the mitogenome of *F. globisporum*. Tandem repeat sequences accounted for 0.35% and 0.14% of the *F. wieringae* and *F. globisporum* mitogenomes, respectively.

### Genetic distance and evolutionary rates of core genes

**Fig. 4:**
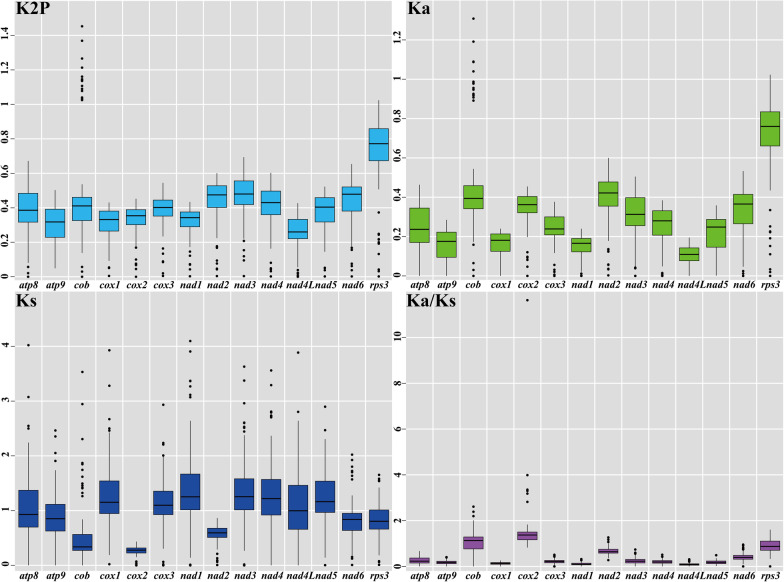
Genetic analysis of 15 protein coding genes conserved in 17mitogenomesfrom *Agaricomycotina*, *Pucciniomycotina* and *Ustilaginomycotina*. K2P, the Kimura-2-parameter distance; *Ka*, the number of nonsynonymous substitutions per nonsynonymous site; *Ks*, the number of synonymous substitutions per synonymous site

Among the 15 detected core PCGs, the *rps3* gene had the largest average Kimura-2-parameter (K2P) genetic distance between the 17 species from *Agaricomycotina*, *Pucciniomycotina*, and *Ustilaginomycotina*, followed by the *nad3* and *nad6* genes, demonstrating that these genes had differentiated greatly during evolution (Fig. 4). The *nad4L* gene exhibited the smallest K2P genetic distance between the 17 species from *Agaricomycotina*, *Pucciniomycotina*, and *Ustilaginomycotina*, indicating that this gene was highly conserved. The *rps3* gene exhibited the largest non-synonymous substitutions rate (*Ka*) among the 15 detected core PCGs, while *nad4L* had the smallest *Ka* value. The synonymous substitution rate (*Ks*) of the *nad1* gene was largest, while that of the *cox2* gene was smallest among the 17 species from *Agaricomycotina*, *Pucciniomycotina*, and *Ustilaginomycotina*. The *Ka/Ks* values for most of the 15 core PCGs were < 1, indicating that these genes were subjected to purifying selection pressure. However, the average *Ka/Ks* values of the *cob*, *cox2*, *nad2*, and *rps3* genes were > 1, indicating these genes might have been subjected to positive selection.

### Intron dynamics of cox1 genes

**Fig. 5:**
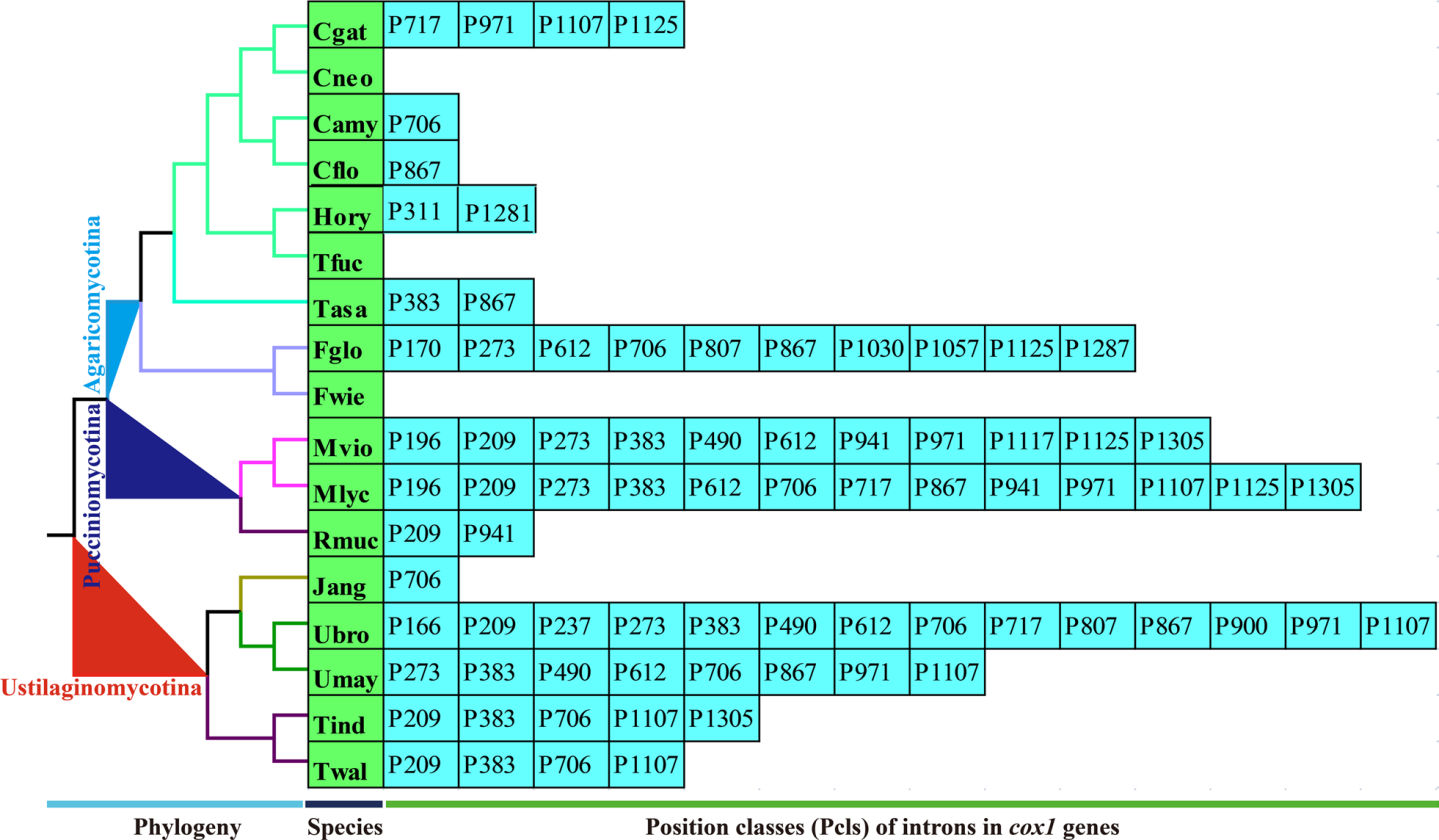
Position class (Pcl) information of *cox1* genes. Introns in *cox1* genes of 17 mitogenomes from *Agaricomycotina*, *Pucciniomycotina* and *Ustilaginomycotina* were classified into different position classes (Pcls) using the *cox1* gene of *Ganoderma calidophilum* as the reference. Each Pcl was constituted by introns inserted at the same position of corresponding *cox1* gene and named according to its insertion site in the aligned corresponding reference sequence (nt). The Pcls present in more than 1/5 of the 17 mitogenomes were considered as common Pcls, while introns detected in less than 1/5 of species were considered to be rare introns. Phylogenetic positions of the 17 species were established using the Bayesian inference (BI) method and Maximum Likelihood (ML) method based on concatenated mitochondrial genes. Species information is shown in Additional file 1: Table S7

A total of 197 introns were detected in the in the *rns*, *rnl*, *atp6*, *atp9*, *cob*, *cox1*, *cox2*, *cox3*, *nad1*, *nad3*, and *nad5* genes of the 17 mitogenomes from *Agaricomycotina*, *Pucciniomycotina* and *Ustilaginomycotina* (Additional file 1: Table S1). The *cox1* gene was the largest host gene of mitochondrial introns, harboring 78 introns accounting for 39.59% of the total introns in mitogenomes from *Agaricomycotina*, *Pucciniomycotina*, and *Ustilaginomycotina*. Therefore, the intron dynamics in the *cox1* gene could significantly influence mitogenome size and organization. Introns in the *cox1* genes of the 17 mitogenomes were classified into different position classes (Pcls) using the *cox1* gene of the medical fungus *Ganoderma calidophilum* (Li et al. 2019d) as a reference. The same Pcl genes from different species were considered to be orthologous introns. The 78 introns in *cox1* genes of the 17 mitogenomes were classified into 25 Pcls (Fig. 5). The class and number of introns in different species varied greatly, indicating potential intron loss/gain events. Pcls present in more than one-fifth of species were considered to be common introns, while others were considered rare introns. In the present study, nine common Pcls and 16 rare Pcls were detected in the 17 mitogenomes from *Agaricomycotina*, *Pucciniomycotina*, and *Ustilaginomycotina*. The most widely distributed intron was P706, which was detected in eight of the 17 species. Intron P383 was the second most common intron, being distributed in seven of the 17 mitogenomes. Several rare Pcls (P166, P170, P237, P311, P900, P1030, P1057, P1117, P1281, and P1287) were only detected in one of the 17 species. However, some rare introns in *Agaricomycotina*, *Pucciniomycotina*, and *Ustilaginomycotina*, including P237, P900, P1030, and P1057, were detected in distantly related species, such as *Hygrophorus russula* (Li et al. 2019c), *Pleurotus citrinopileatus* (Li et al. 2018a), *Armillaria solidipes* (Kolesnikova et al. 2019), and *Rhizopogon salebrosus* (Li et al. 2019a) from *Agaricomycetes*, indicating possible gene transfer events. P166, P170, P311, P1117, P1281, and P1287 were only detected in *Agaricomycotina*, *Pucciniomycotina*, and *Ustilaginomycotina* species, and no homologous introns were found from other Basidiomycota species. The *cox1* gene of *F. globisporum* contained 10 Pcls, while no introns were identified in the *cox1* gene of *F. wieringae*. These results indicated that the ancestors of *Filobasidium* species lost or gained introns on a large-scale during evolution.

### Gene arrangement and comparative mitogenomic analysis

**Fig. 6:**
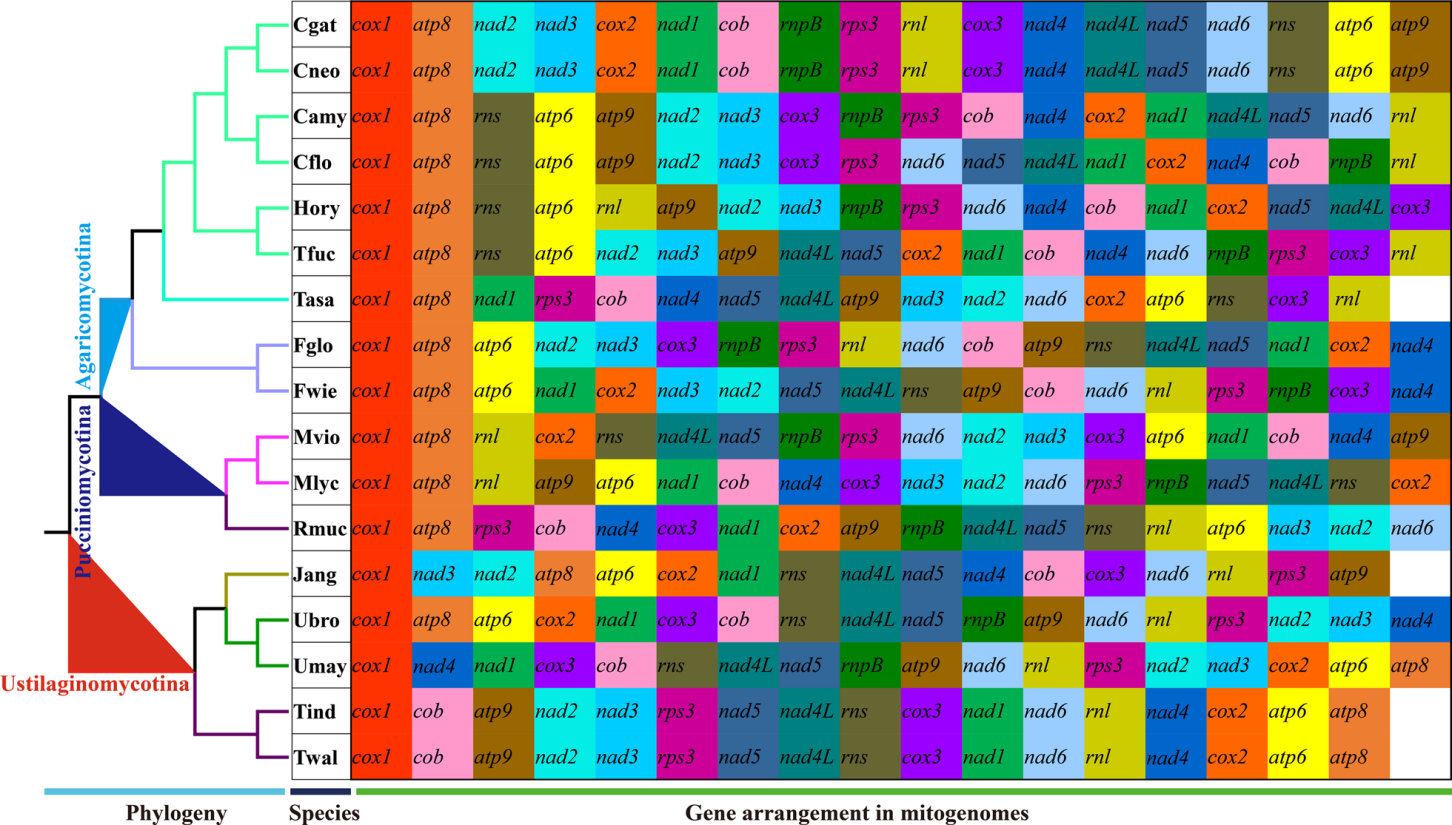
Mitochondrial gene arrangement analyses of 17 mitochondrial genomes from *Agaricomycotina*, *Pucciniomycotina* and *Ustilaginomycotina*. The same gene were represented by same color blocks. Phylogenetic positions of the 17 species were established using the Bayesian inference (BI) method and Maximum Likelihood (ML) method based on concatenated mitochondrial genes. Species information is shown in Additional file 1: Table S7

In the present study, we analyzed mitochondrial gene arrangements, including 15 core PCGs and two rRNA genes, of the 17 species from *Agaricomycotina*, *Pucciniomycotina*, and *Ustilaginomycotina* (Fig. 6). The results showed that the mitochondrial gene arrangements varied greatly between species. Large-scale gene rearrangements were detected between species from different genera, including gene relocations, and position exchanges. We also observed several gene rearrangements between species from the same genera, such as *Cryptococcus neoformans* and *Cryptococcus amylolentus*, *Microbotryum cf. violaceum* and *Microbotryum lychnidisdioicae*, and *Ustilago bromivora* and *Ustilago maydis*. The same gene arrangements were only observed between *Cryptococcus gattii* and *Cryptococcus neoformans*, as well as between *Tilletia indica* and *Tilletia walker*, which had a close phylogenetic relationship. Large-scale gene rearrangements have also occurred in the two *Filobasidium* mitogenomes, and 13 of the 17 mitochondrial genes have undergone positional changes.

The sizes of 17 mitogenomes tested varied greatly, ranging from 24,874 to 177,540 bp, with an average size of 46,800 bp (Additional file 1: Table S1). The mitogenome of *F. globisporum* (71,783 bp) was the fourth largest among the 17 mitogenomes from *Agaricomycotina*, *Pucciniomycotina*, and *Ustilaginomycotina*, which was only smaller than that of *Ustilagobromivora* (177,540 bp, acc. LT558140 in the NCBI database) from the order *Ustilaginales*, *Microbotryum cf. violaceum* (92,107 bp), and *Microbotryum lychnidisdioicae* (107,808 bp) from the order *Microbotryales*. The GC content of the 17 mitogenomes ranged from 28.79 to 40.43%, with an average of 34.15%. The GC content of the two *Filobasidium* species was much higher than the average value. Eight and five of the 17 mitogenomes from *Agaricomycotina*, *Pucciniomycotina*, and *Ustilaginomycotina* had positive AT skews and GC skews, respectively. Each mitogenome contained 15–51 PCGs, and the mitogenome of *Microbotryum lychnidisdioicae* contained the most PCGs. The mitogenome of *F. globisporum* contained the greatest number of introns (37) and intronic ORFs (26) among the 17 mitogenomes detected. All 17 mitogenomes contained two rRNA genes. In addition, 20–31 tRNA genes were detected in the17 species from *Agaricomycotina*, *Pucciniomycotina* and *Ustilaginomycotina*.

### Phylogenetic analysis

**Fig. 7:**
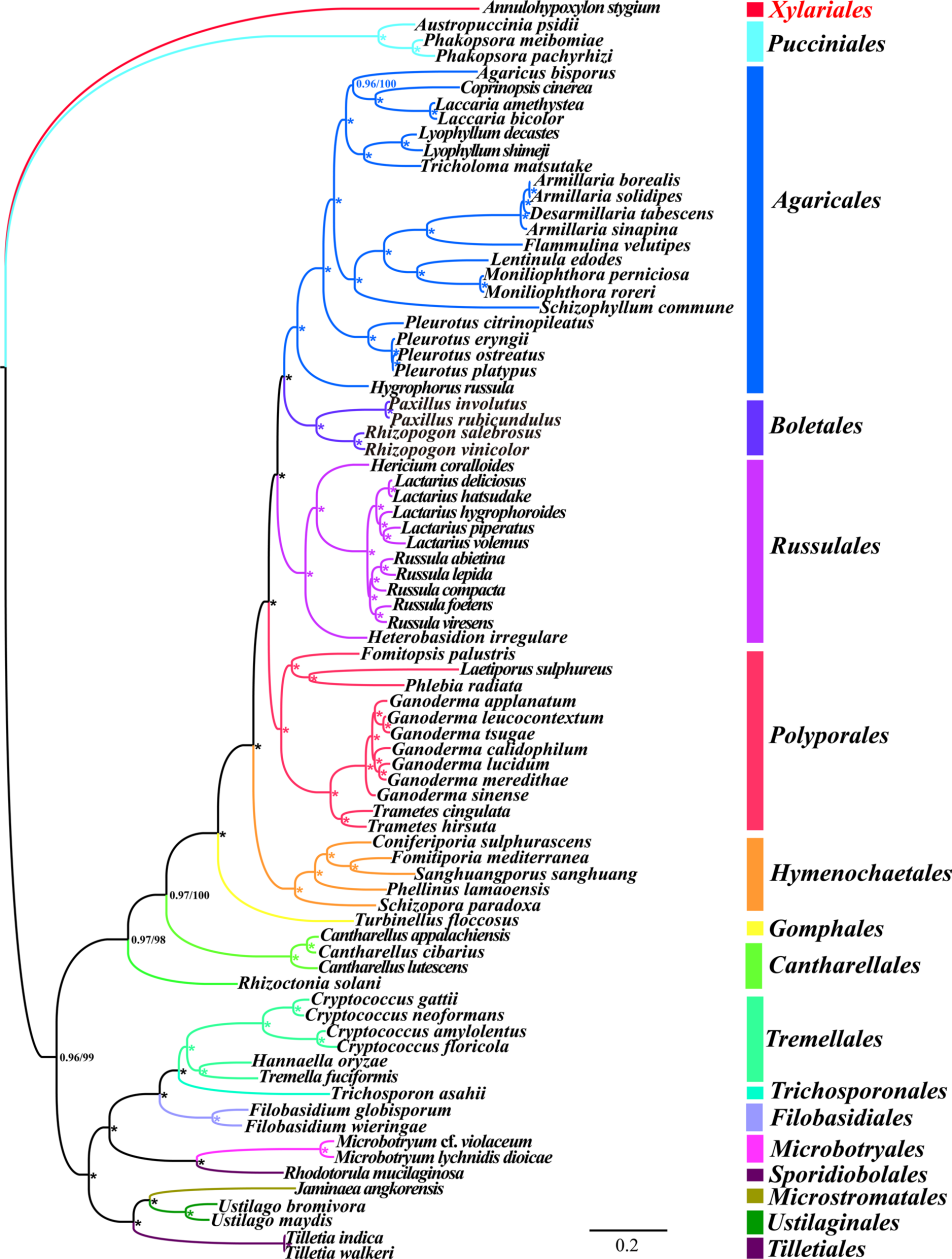
Molecular phylogeny of 79Basidiomycota species based on Bayesian inference (BI) and Maximum likelihood (ML) analysis of 15 protein coding genes. Support values are Bayesian posterior probabilities (before slash) and bootstrap (BS) values (after slash).The asterisk on the evolutionary clades indicates that the BPP value is 1 and the BS value is 100.Species and NCBI accession numbers for mitogenomes used in the phylogenetic analysis are provided in Additional file 1: Table S7

We obtained an identical and well-supported phylogenetic tree based on mitochondrial gene sets using both BI and ML methods (15 core PCGs) (Fig. 7). All major clades within the phylogenetic tree were well supported (BPP ≥ 0.96; BS ≥ 98). According to the phylogenetic tree, the 79 Basidiomycota species could be divided into 16 major clades corresponding to the orders *Agaricales*, *Boletales*, *Cantharellales*, *Filobasidiales*, *Gomphales*, *Hymenochaetales*, *Microbotryales, Microstromatales, Polyporales, Pucciniales, Russulales, Sporidiobolales, Tilletiales, Tremellales, Trichosporonales,* and *Ustilaginales* (Additional file 1: Table S7). Phylogenetic analysis indicated that the two *Filobasidium* species branched basally to the other two *Tremellomycete* orders (*Trichosporonales* and *Tremellales*) (Liu et al. 2015a, b). Phylogenetic analysis based on the mitochondrial gene set also showed that the mitogenome was an effective molecular marker for analysis of the phylogenetic relationship of basidiomycetes.

## DISCUSSION

The mitogenome was obtained from endosymbiotic bacteria by the ancestors of eukaryotes (Gray et al. 2001). During the long-term evolution and differentiation of eukaryotes, the mitogenomes of most eukaryotes contracted. Many ancient mitochondrial genes have been transferred into the nuclear genome, which is considered to have several advantages (Adams and Palmer 2003; Adams et al. 2002). However, a small number of mitochondrial genes have been retained, including a set of core PCGs for energy metabolism, two rRNA genes, and 5–35 tRNA genes (Allen 2015; Wang et al. 2020a). These retained genes play important roles in regulation of cell homeostasis and mitochondrial function (Allen 2015; Bjorkholm et al. 2015). In the present study, we found that the core PCGs of the two *Filobasidium* species varied in length and base composition. In addition, possible positive selections on *cob*, *cox2*, *nad2*, and *rps3* genes were detected between some species from *Agaricomycotina*, *Pucciniomycotina*, and *Ustilaginomycotina*. Core PCGs, including *atp6*, *atp8*, *atp9*, *cob*, *cox1*, *cox2*, *cox3*, *nad1*, *nad2*, *nad3*, *nad4*, *nad4L*, *nad5*, and *nad6*, are used for energy metabolism, and the *rps3* gene is likely involved in assembly of the mitochondrial small (37S) ribosomal subunit (Seif et al. 2005). The effects of size and length variations of these genes on fungal phenotypes need to be further verified. *Tremellomycetes* species have diverse lifestyles and morphological characteristics. Some species are parasitic, some are saprophytic, some are symbiotic, some can form basidiocarps, and some are yeast like (Millanes et al. 2011; Yurkov and Kurtzman 2019). This diversity may result in positive selection pressure on core PCGs of *Tremellomycetes* mitogenomes. In addition, the lengths, and base compositions of rRNA genes and tRNA genes in two *Filobasidium* species also varied in this study. Previous studies have shown that the base mutation of mitochondrial tRNA can affect protein synthesis (Ding et al. 2019; Lin et al. 2019); however, the effects of rRNA and tRNA variations on the growth, development and physiological activities of *Filobasidium* species need to be further analyzed.

In the present study, large mitogenome size variations were detected between two *Filobasidium* species. Specifically, the mitogenome of *F. globisporum* was 2.58 times greater than that of *F. wieringae*. The intronic region was considered to be the most important factor leading to size expansion of the *F. globisporum* mitogenome, with a contribution rate of 81.48%. These results were consistent with those of previous studies, suggesting that introns played an important role in the size variations of fungal mitogenomes (Li et al. 2020c; Ye et al. 2020). Introns are considered mobile genetic elements in the fungal mitogenome, and changes in their dynamics have a significant impact on the size and organization of the fungal mitogenome (Hamari et al. 2002; Repar and Warnecke 2017; Sandor et al. 2018). In the present study, intron classes and numbers varied greatly between the 17 species from *Agaricomycotina*, *Pucciniomycotina*, and *Ustilaginomycotina*, indicating that frequent intron loss/gain events occurred during evolution of the species. Some introns were observed to be widely distributed in species from *Agaricomycotina*, *Pucciniomycotina*, and *Ustilaginomycotina*, including P209, P273, P383, P612, P706, P867, P1107, and P1125. Interestingly, P273 was considered a rare intron in Basidiomycete species, indicating that the different intron classes were unevenly distributed in basidiomycetes (Ye et al. 2020). Some introns were detected in only one of the 17 species, while homologous introns were detected in distant species from *Agaricomycetes*, indicating potential gene transfer events. In addition, some rare introns were detected only in *Tremellomycetes*, and no homologous introns were detected in other basidiomycete species. Further studies are needed to reveal the origin and evolution of these rare introns in *Tremellomycetes* species to clarify the functions of mobile genetic elements in mitochondria.

The arrangement of mitochondrial genes can be used as an important reference to reflect the phylogenetic status and genetic relationship of species (Li et al. 2018a, b; Wang et al. 2020b). In the present study, we found that the mitochondrial gene arrangement varied greatly in *Tremellomycetes*, and that species from different families had different gene arrangements. In addition, large-scale gene rearrangements were observed between species from the same genera in *Tremellomycetes*, indicating that the orders of mitochondrial genes in *Tremellomycetes* species were highly variable in evolution. The rearrangement of fungal mitochondrial genes has been less studied than that of animal mitochondrial genes. Several models have been proposed to reveal mitochondrial gene rearrangement in animals (Lavrov et al. 2002; Xia et al. 2016); however, the mechanism of mitochondrial gene rearrangement in fungi has not been revealed. Higher repeat sequences than animal mitogenomes may be one of the reasons for frequent rearrangements of fungal mitogenomes (Aguileta et al. 2014).

Basidiomycetes are a diverse group that are distributed worldwide. Basidiomycetes play an important role in industry, medicine, agriculture, and ecological maintenance (Alves et al. 2013; Elisashvili 2012; Voriskova and Baldrian 2013). Accurate classification and identification of basidiomycetes will contribute to their efficient utilization (Hibbett et al. 2007; James et al. 2006). However, some basidiomycetes have limited and overlapping morphological characteristics, which makes them difficult to classify and identify based only on morphology. To date, the nuclear genome and molecular markers have been used for classification of basidiomycetes (Hibbett 2006; James et al. 2006; Spatafora et al. 2016). However, the mitogenome of basidiomycetes is easier to obtain than the nuclear genome and contains more genetic information than individual molecular markers. These advantages make the mitogenome a potential tool for the phylogeny and classification of basidiomycetes (Li et al. 2019b, 2021b, 2020d). However, basidiomycete mitogenomes have been less studied than animal and *Ascomycetes* mitogenomes, with less than 120 complete mitogenomes of basidiomycetes published in the NCBI database. Moreover, the mitogenome of the order *Filobasidiales* has not previously been reported. In the present study, we obtained a phylogenetic tree with a good support rate by using the combined mitochondrial gene set (15 core PCGs), indicating that the mitochondrial gene is an effective tool for analysis of the phylogenetic relationship of basidiomycetes. More mitogenomes are needed to promote the classification or identification of basidiomycetes and reconstruct the phylogeny of fungi.

## CONCLUSIONS

In the present study, two complete mitogenomes from the *Filobasidiales* order were reported and compared with other mitogenomes from *Agaricomycotina*, *Pucciniomycotina*, and *Ustilaginomycotina*, including *F. wieringae* and *F. globisporum*. The mitochondrial genome size of the two *Filobasidium* species varied greatly, ranging from 27,861 to 71,783 bp, and the intronic region was considered to be the main factor contributing to mitogenome size variations in the *Filobasidium* genus. We further found intron loss/gain events in *Tremellomycetes* and other mitogenomes occurred during evolution. Comparative mitogenomic analysis revealed that the genetic contents, codon usages, and repetitive sequence differentiated greatly in the two *Filobasidium* species. In addition, a large number of base and sequence length variations were found in the core coding genes, tRNA genes and rRNA genes of the two *Filobasidium* mitogenomes. Several core PCGs have experienced strong pressure of positive selection in mitogenomes from *Agaricomycotina*, *Pucciniomycotina*, and *Ustilaginomycotina*, including *cob*, *cox2*, *nad2*, and *rps3* genes. In addition, large-scale gene rearrangements were detected between the 17 species from *Agaricomycotina*, *Pucciniomycotina*, and *Ustilaginomycotina*, which showed that the mitochondrial gene arrangement was highly variable. We further analyzed the phylogenetic status of *Filobasidium* species based on BI and ML methods using a combined mitochondrial gene set. This study serves as the first investigation of mitogenomes from the order *Filobasidiales*, and the results presented herein will help improve our understanding of *Filobasidiales* genomics, evolution, and taxonomy.

## Supplementary Information

**Additional file 1: Table S1**. Comparison on mitogenomes among 17 species from *Agaricomycotina*, *Pucciniomycotina* and *Ustilaginomycotina*. **Table S2**. Annotation and characterization of the two *Filobasidium mitogenomes*. **Table S3**. Core protein coding gene information of the two *Filobasidium* species. **Table S4**. Start and stop codons analysis of 17 species from *Agaricomycotina*, *Pucciniomycotina* and *Ustilaginomycotina*. **Table S5**. Local BLAST analysis of the *Filobasidium mitogenomes* against themselves. **Table S6**. Tandem repeats detected in the mitogenomes of *Filobasidium* using the online program Tandem Repeats Finder. **Table S7**. Species, and GenBank accession number used for phylogenetic analysis in this study.

**Additional file 2: Figure S1.** Putative secondary structures of tRNA genes identified in the mitochondrial genomes of two *Filobasidium* species. The 22 tRNAs in green or red fonts represent tRNAs shared by the two *Filobasidium* species, while the tRNA in blue font represent tRNA only in *F. wieringae*. Residues conserved across the two mitochondrial genomes are shown in green, while variable sites are shown in red. All genes are shown in order of occurrence in the mitochondrial genome of *F. wieringae*, starting from *trnL*.

## Data Availability

All data generated or analyzed during this study are included in this published article [and its supplementary information files].

## Abbreviations

Mitogenome: Mitochondrial genome
PCG: Protein-coding gene
Pcls: Position classes
*Ks*: Synonymous substitution rates
*Ka*: Nonsynonymous substitution rates
BI: Bayesian inference
ML: Maximum likelihood
