# Supplementary figures and images for: First two mitochondrial genomes for the order Filobasidiales reveal novel gene rearrangements and intron dynamics of Tremellomycetes
Source: IMA Fungus. 2022 May 2;13:7. doi: 10.1186/s43008-022-00094-2 (PMC9059411; doi:10.1186/s43008-022-00094-2)

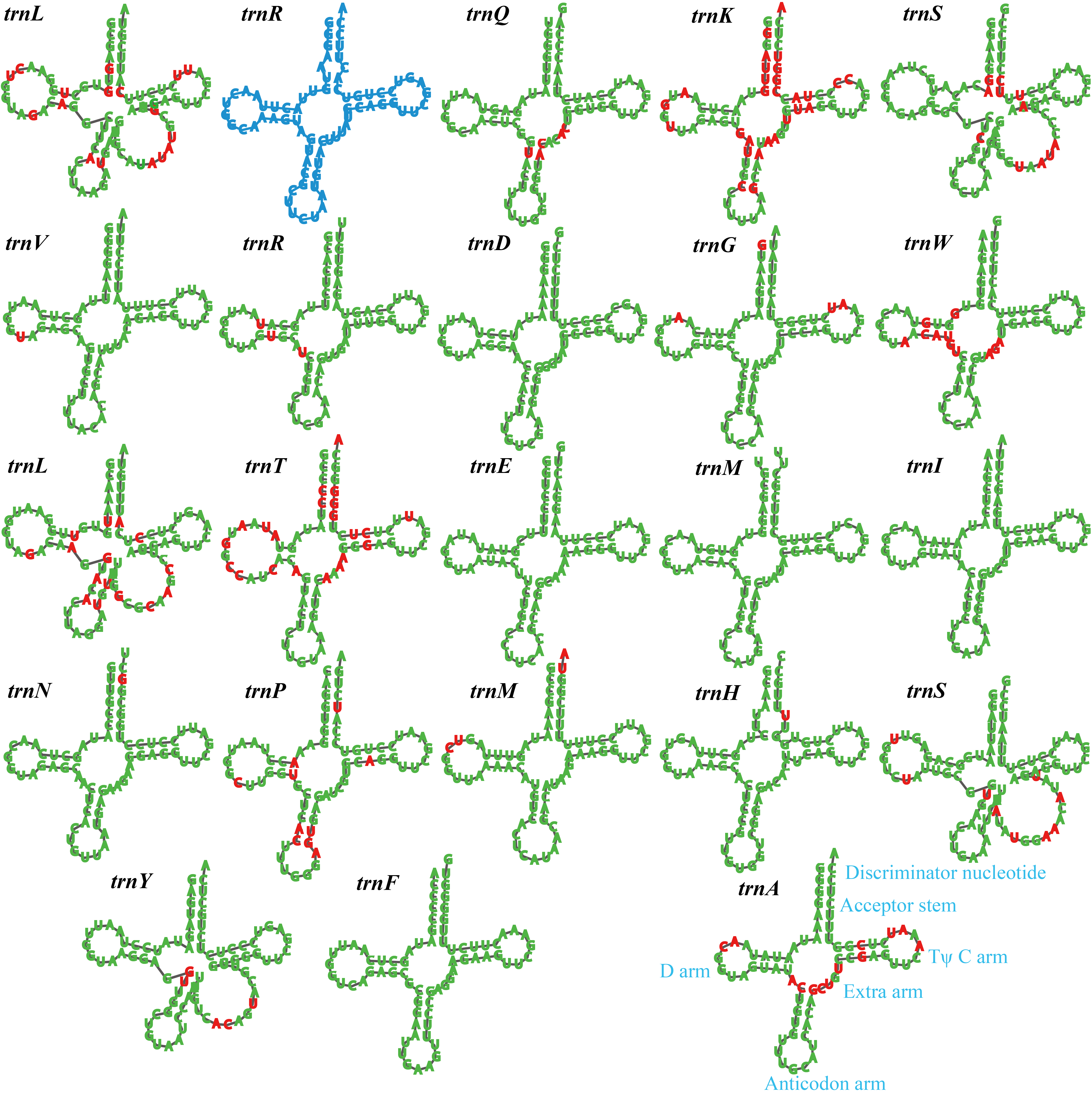

Supplement: Supplementary file 2 — Additional file 2: Figure S1. Putative secondary structures of tRNA genes identified in the mitochondrial genomes of two Filobasidium species. The 22 tRNAs in green or red fonts represent tRNAs shared by the two Filobasidium species, while the tRNA in blue font represent tRNA only in F. wieringae. Residues conserved across the two mitochondrial genomes are shown in green, while variable sites are shown in red. All genes are shown in order of occurrence in the mitochondrial genome of F. wieringae, starting from trnL. [file 43008_2022_94_MOESM2_ESM.tif]
